# Synapse development is regulated by microglial THIK-1 K^+^ channels

**DOI:** 10.1073/pnas.2106294118

**Published:** 2021-10-12

**Authors:** Pablo Izquierdo, Hiroko Shiina, Chanawee Hirunpattarasilp, Grace Gillis, David Attwell

**Affiliations:** ^a^Department of Neuroscience, Physiology and Pharmacology, University College London, London WC1E 6BT, United Kingdom

**Keywords:** microglia, phagocytosis, synaptic pruning

## Abstract

Microglia are the resident immune cells of the central nervous system. They constantly survey the brain parenchyma for redundant synapses, debris, or dying cells, which they remove through phagocytosis. Microglial ramification, motility, and cytokine release are regulated by tonically active THIK-1 K^+^ channels on the microglial plasma membrane. Here, we examined whether these channels also play a role in phagocytosis. Using pharmacological blockers and THIK-1 knockout (KO) mice, we found that a lack of THIK-1 activity approximately halved both microglial phagocytosis and marker levels for the lysosomes that degrade phagocytically removed material. These changes may reflect a decrease of intracellular [Ca^2+^]_i_ activity, which was observed when THIK-1 activity was reduced, since buffering [Ca^2+^]_i_ reduced phagocytosis. Less phagocytosis is expected to result in impaired pruning of synapses. In the hippocampus, mice lacking THIK-1 expression had an increased number of anatomically and electrophysiologically defined glutamatergic synapses during development. This resulted from an increased number of presynaptic terminals, caused by impaired removal by THIK-1 KO microglia. The dependence of synapse number on THIK-1 K^+^ channels, which control microglial surveillance and phagocytic ability, implies that changes in the THIK-1 expression level in disease states may contribute to altering neural circuit function.

## Significance

Microglia are the brain’s resident immune cells, surveying the brain with motile processes, which can remove pathogens but also prune unnecessary junctions between the neurons (synapses). A potassium channel, THIK-1, in the microglial membrane allows efflux of potassium from these cells and thereby regulates their membrane voltage as well as their process motility and release of inflammatory mediators. Here, using THIK-1–blocking drugs and THIK-1–deficient mice, we demonstrate that THIK-1 controls removal of synaptic material by microglia, which reduces the number of functional synapses in the developing brain.

Microglia are the primary immune cells in the brain parenchyma, accounting for 5 to 12% of all cells, with a particularly high density in the hippocampus (1). Their functions are controlled by a plethora of ion channels and receptors on their plasma membrane (2). Even so-called “resting” microglia are constantly scanning the surrounding brain tissue by extending and retracting their processes. Baseline surveillance is regulated independently of targeted chemotaxis and is controlled by membrane voltage, the resting value of which (around −40 mV) is set by the two-pore domain halothane-inhibited K^+^ (THIK-1) channel, the main K^+^ channel expressed by microglia in situ (3).

Microglia can internalize and degrade targets that they have previously detected and contacted. This process, referred to as phagocytosis, occurs throughout life in both physiological and pathological conditions. Microglia can phagocytose both dead and viable cells (4), myelin sheaths (5), and amyloid debris (6).

However, microglia are more than mere cleaners of the brainparenchyma. These cells are key for normal brain development (7), and their processes interact with various neuronal compartments. While microglial contacts at nonsynaptic regions regulate dendrite-branching and axonal pathfinding during development as well as neuronal excitability, contacts at synaptic sites control synapse formation, strength, plasticity, and elimination (8–10). Microglia–synapse contacts can lead to formation of dendritic spines as well as new filopodia in spine heads (11, 12). On the other hand, microglia-mediated circuit pruning also occurs as redundant or less-active synapses are removed (13, 14). Synapse phagocytosis is controlled by a fine balance between “eat-me” and “don't-eat-me” signals, which respectively promote removal (e.g., C1q or C3/CD11b) (15) or protect synapses from pruning (e.g., CD47) (16). A number of signaling pathways are involved in microglia-mediated removal of synapses during development, including complement factors in the visual system (14, 17) or the fractalkine receptor (13, 18) and TREM2 (19) in the hippocampus.

Microglial THIK-1 K^+^ channels maintain ramification of microglial processes and their surveillance of the brain (3), which could promote the elimination of synapses during development by phagocytosis (or trogocytosis) (12). Immune cells have been shown to hyperpolarize when phagocytosing targets (20), so in addition to promoting microglial-synapse interactions, the hyperpolarization maintained by microglial THIK-1 channels might also promote phagocytosis directly. Regulation of phagocytosis by membrane potential could also explain why phagocytosis is impaired in TREM2 knockout mice in which microglia are depolarized (21).

Here, we identify membrane K^+^ flux as a factor regulating microglial phagocytosis and show that block or deletion of THIK-1 channels reduces microglial phagocytosis in situ. Intracellular calcium activity (which regulates phagocytosis) and lysosome marker levels are also reduced in microglia when THIK-1 channel function is absent. THIK-1 deletion results in decreased microglial uptake of presynaptic material in the developing hippocampus and thus evokes an increase in the number of functional glutamatergic synapses. Because developmental mechanisms are thought to be reactivated during aging and disease (22–26), understanding these mechanisms controlling microglial phagocytosis may have therapeutic value in the mature brain.

## Materials and Methods

### Rodent Procedures.

All animal procedures were performed in accordance with the UK Animals (Scientific Procedures) Act 1986 (Home Office License 70/8976) after ethical review by the University College London Animal Welfare and Ethical Review Body. Rodents were maintained on a 12-h/12-h light/dark cycle, and food and water were available ad libitum. Mice (postnatal day P17 to 19, P26 to 32, and P120 to 130) were housed in individually ventilated cages, and Sprague–Dawley rats (P12 to 13) were kept in open-shelf units. Animals of both sexes were euthanized by cervical dislocation followed by decapitation or by an overdose of pentobarbital sodium (Euthatal, 200 µg/g body weight) injected intraperitoneally before transcardial perfusion fixation of tissue with 4% paraformaldehyde (PFA).

For mouse experiments, THIK-1 knockouts (Kcnk13-IN1-EM1-B6N) were generated by Medical Research Council Harwell as previously described in detail (3). Briefly, a single nucleotide insertion in the gene encoding the THIK-1 channel protein (*Kcnk13*) leads to a premature stop codon, producing a truncated protein that fails to form a channel.

For Ca^2+^ imaging, GCaMP5g-IRES-tdTomato transgenic mice (27) were crossed with *Cx3cr1*^CreER^ mice (28), which allowed expression of GCaMP5g and tdTomato in microglia at least 21 d following tamoxifen gavage (Sigma T5648, 120 µg/g body weight for 4 consecutive days). Only *Cx3cr1* heterozygotes were used for experiments.

### Acute Brain Slicing.

Dorsal hippocampal slices (parasagittal, 250 µm) were prepared on a Leica VT1200S vibratome in ice-chilled slicing solution containing the following (in millimolar): 124 NaCl, 2.5 KCl, 26 NaHCO_3_, 1 NaH_2_PO_4_, 10 glucose, 1 CaCl_2_, 2 MgCl_2_, and 1 kynurenic acid. Osmolarity was adjusted to ∼295 mOsM and pH set to 7.4 when bubbled with 5% CO_2_.

For electrophysiology experiments, 300-µm parasagittal hippocampal slices from mice were prepared in ice-cold N-methyl-D-glucamine (NMDG)–based slicing solution (to reduce cell swelling), containing the following (in millimolar): 93 NMDG, 2.5 KCl, 20 HEPES, 30 NaHCO_3_, 1.2 NaH_2_PO_4_, 25 glucose, 0.5CaCl_2_, 10 MgCl_2_, 5 sodium ascorbate, 2.4 sodium pyruvate, and 1 kynurenic acid. The osmolarity was adjusted to ∼300 mOsM and pH set to 7.4. Slices were immediately transferred to warmed (35 °C) slicing solution for 20 min and then to storage solution at room temperature containing the following (in millimolar): 92 NaCl, 2.5 KCl, 20 HEPES, 30 NaHCO_3_, 1.2 NaH_2_PO_4_, 25 glucose, 2 CaCl_2_, 1 MgCl_2_, 5 sodium ascorbate, 2.4 sodium pyruvate, and 1kynurenic acid. The osmolarity was adjusted to ∼300 mOsm, and pH set to 7.4.

### Microbead Phagocytosis Assay.

Hippocampal brain slices were allowed to recover at room temperature for 2.5 h so that the microglia would activate and become more phagocytic (29–31). They were then transferred to 24-well plates and incubated with 3 µm serum-coated, fluorescein isothiocyanate (FITC)-labeled resin microbeads (Sigma 72439; 1.7 × 10^7^ beads per well in artificial cerebrospinal fluid [aCSF]) for 1.5 h in a cell culture incubator at 35 °C (or at 4 °C, as a negative control). Bead suspensions were supplemented as indicated with the following drugs: bupivacaine (Sigma B5274), charybdotoxin (Anorspec 28244), cytochalasin D (Sigma 22144), MRS2578 (Cayman CAY19704), tetrapentylammonium (TPA, Sigma 258962), or tetrodotoxin citrate (TTX; Abcam ab120055). In addition, 30 min prior to applying the bead suspension (i.e., during the last 30 min of the recovery period), slices were preincubated with the drugs at the same concentration as that contained in the bead suspension. For high [K^+^]_o_, aCSF containing 120 mM KCl (replacing 117.5 mM NaCl with KCl) was used. While not every drug could be tested on slices from every animal (a larger number of slices than it is possible to obtain from a single animal was required to test all the drugs used in the experiments), all experiments included untreated (control) slices to confirm that replicates from different animals were comparable. Following incubation, slices were rinsed in cold phosphate-buffered saline (PBS), fixed in 4% PFA for 45 min at room temperature, and immunostained as described in *Immunohistochemistry*. Confocal stacks were obtained at 2-μm z-step intervals using a Zeiss LSM700 microscope with a Plan-Apochromat 20×/0.8 objective. To assess phagocytosis, the percentage of phagocytic microglia (i.e., Iba1^+^ cells which had internalized >1 FITC^+^ beads) was calculated. A total of five to 26 slices from three to 10 animals were used per condition. Data points shown on plots indicate individual animal averages. Data from slices coming from different animals were not significantly different. All imaging and analyses were done with the researcher blind to genotype and treatment.

### Immunohistochemistry.

Free-floating 250-µm hippocampal slices were permeabilized and blocked for 2 h at room temperature in a solution containing 10% normal horse serum and 0.02% Triton X-100 in PBS followed by incubation with primary antibodies in blocking buffer for 12 h at 4 °C with agitation (mouse anti-Bassoon [1:300, Novus NB120-13249], rabbit anti-Homer1 [1:300, Synaptic Systems 160002], and rabbit anti-Iba1 [1:500, Synaptic Systems 234003]). Following four 10-min washes in PBS, secondary antibodies diluted 1:1,000 in blocking buffer (donkey anti-mouse Alexa 488, donkey anti-rabbit Alexa Fluor 555 or 647; Invitrogen) were applied for 4 h at room temperature with agitation. Finally, slices were washed in PBS and mounted.

### Western Blotting.

Hippocampi from P17 mice were dissected and immediately placed in dry ice. Protein homogenates were prepared in radioimmunoprecipitation assay (RIPA) lysis buffer containing 50 mM Tris pH 7.5, 1 mM ethylene diamine tetraacetic acid, 2 mM ethylene glycol tetraacetic acid (EGTA), 150 mM NaCl, 1% NP-40, 0.5% sodium deoxycholate, 0.1% sodium dodecyl sulfate, and one tablet of protease inhibitor cocktail (Roche 11836153001) per 10 mL of buffer. Samples were homogenized on ice, centrifuged for 30 min at 12,000 rpm at 4 °C, and protein concentration in the supernatant was determined with a Pierce bicinchoninic acid (BCA) protein assay kit (Thermo Fisher Scientific 232227). Samples (30 µg) were loaded onto 4%/12% acrylamide gels and resolved by electrophoresis. Proteins were transferred to 0.45-µm nitrocellulose membranes (GE 10600002), which were incubated in blocking solution containing 0.1% Tween-20 and 4% powder milk (wt/vol) in PBS for 1 h at room temperature. Immunoblotting followed in sealed plastic film bags (rabbit anti-CD68 [1:500, Abcam ab125212] and rat anti-LAMP1 [1:500, Millipore MABC39]) overnight at 4 °C. Following three 10-min washes in 0.1% Tween-20 in PBS, membranes were incubated for 1 h at room temperature in horseradish peroxidase–conjugated secondary antibodies diluted 1:10,000 in blocking solution. Following three 10-min washes, signals were detected with chemiluminescent substrate (Thermo Fisher 34075) with an ImageQuant LAS 4000 camera. Bands were quantified using the Gels tool in ImageJ/FIJI, and values were normalized for loading using β-actin (1:5,000, Proteintech 66009).

### Microglial Ca^2+^ Imaging.

Hippocampal brain slices from *Cx3cr1*^CreER^ × GCaMP5g-IRES-tdTomato mice were imaged on a Zeiss LSM780 two-photon microscope with a Plan-Apochromat 20×/1.0 objective. An area of 106 µm × 106 µm was scanned (pixel size 0.21 µm; 1-µs pixel dwell time; overall frame acquisition time 25 ms) with the laser tuned to 920 nm to allow acquisition of both GCaMP5g and tdTomato signals. Prior to imaging, a z-stack (1-µm steps) was taken to confirm the morphology of the imaged microglia as visualized by tdTomato. Then, single-plane images were acquired every second for 5 min (300 frames) to minimize bleaching, with drugs added to the circulating aCSF 10 min prior to recording where indicated.

To examine lesion-evoked [Ca^2+^]_i_ rises, a 20-s baseline was recorded, after which a laser injury was performed in an area >30 µm from microglial cells and a further 5 min of images (300 frames) were recorded. For the injury, a circular region of interest (ROI; 6-µm radius) was drawn, which was bleached in a single scan (177.3-µs pixel dwell time) with the laser tuned to 920 nm at 80% of its maximum power.

For analysis, all movies from the same field of view were concatenated and registered using the MultiStackReg and TemplateMatching tools in ImageJ/FIJI. No further image processing was done. ROIs were then drawn around microglial somata and ΔF/F was calculated as (F_t_ − F_o_)/F_o_, where F_t_ is the fluorescence intensity of the ROI at each timepoint and F_o_ is the fluorescence averaged over the baseline.

For spontaneous Ca^2+^ analysis, we used custom-written MATLAB code adapted from ref. 32, available from https://github.com/AttwellLab/MyelinCalcium. For each ROI, a locally time-smoothened baseline was generated using a baseline fit with the piecewise cubic Hemite interpolating polynomial in MATLAB, with a smoothing time of 100 frames. Then, Ca^2+^ transients were defined using a detection threshold set to 2.25 × the SD of the first 100 points in the trace that had ΔF/F < 0.1 (to exclude contributions of Ca^2+^ transients to the baseline). Transients were then further confirmed using a minimal area threshold (∫ΔF/F dt> 0.15 [i.e., they were excluded if this condition was not satisfied]) and manually checked to exclude false positives (e.g., resulting from increased background signal). Finally, Ca^2+^ transient rates per 300 s were calculated.

For evoked Ca^2+^ change analysis, ΔF/F was calculated using the fluorescence intensity of the ROI averaged over the 20 s preceding the laser lesion as a baseline. Values of ΔF/F at the peak following the stimulus were compared for statistical analysis. For producing representative images of the GCaMP peak signal, a multicolor (Fire) lookup table was applied to microglial images in ImageJ/FIJI to help distinguish different intensity values.

### Synapse Quantification.

Brain slices were imaged (102 µm × 102 µm) at 3 to 6μm from the slice surface using a Zeiss LSM700 microscope with a Plan-Apochromat 63×/1.4 objective. Three confocal images from the CA1 stratum radiatum region (at 1.5-μm intervals) were taken per brain slice, and five brain slices were taken per animal. Images were analyzed individually and then averaged across stacks and brain slices to obtain animal means. After background subtraction (with a 10-pixel rolling ball average), marker areas were quantified using a custom-based intensity threshold protocol with ImageJ/FIJI, and the Analyze Particles function was used to quantify puncta number and areas. For thresholding, sample images were first manually thresholded for each channel blinded to condition and genotype, and a suitable range above threshold was established, which was then kept constant throughout (Bassoon: 15–255; Homer1: 30–255; images were 8-bit). Size exclusion (>1.2 μm^2^ and <0.05 μm^2^) was applied to exclude any objects unlikely to represent synaptic puncta. Synapses were defined by the presence of overlapping presynaptic–postsynaptic puncta (33). Presynaptic colocalization with microglia was analyzed as the total area of Bassoon puncta within the Iba1-stained cell area after thresholding. All imaging and analysis were done with the researcher blind to genotype or treatment.

### Cell Density Analysis.

Brain slices were imaged (640 µm × 640 µm) using a Zeiss LSM700 microscope with a Plan-Apochromat 20×/0.8 objective. Cell density and spatial distribution were analyzed as in ref. 34. Briefly, cell counts were performed to obtain cell density as well as the average nearest-neighbor distance between cells (NND) and their regularity index. The latter is the ratio of the mean NND to the SD of the NND for the whole population of cells and describes how regular the spacing of microglia is. One maximum-projected z-stack (3-µm depth) from the CA1 stratum radiatum region was analyzed per slice, and five slices were averaged per animal.

### Electrophysiology.

Slices were individually transferred to the recording chamber and perfused at 3 to 5 mL/min with aCSF, which was maintained at 32 to 34 °C. Pyramidal neurons in the CA1 region of the dorsal hippocampus were selected visually using an Olympus 60×/0.9 water-immersion objective in combination with differential interference contrast optics. Cells were recorded in the whole-cell, voltage-clamp configuration with glass patch-pipettes (resistance in the bath solution 2 to 4 MΩ). Junction potentials (−10 mV) were corrected for. Recorded signals were sampled and digitized at 20 kHz, filtered at 4 kHz, and then further filtered offline at 2 kHz for analysis and data presentation. During the entire course of recording, access resistance was monitored by periodically applying a −5-mV voltage pulse. Cells were excluded from analysis if the access resistance changed by more than 20% during the course of an experiment.

To study excitatory synapses, pipettes were filled with internal solution containing the following (in millimolar): 132.3 K-gluconate, 7.7 KCl, 4 NaCl, 0.5 CaCl_2_, 10 HEPES, 5 EGTA, 4 MgATP, and 0.5 Na_2_GTP (pH 7.2 to 7.3). The calculated reversal potential for Cl^−^ (E_Cl_) with these solutions was ∼−62 mV, and a holding potential (V_h_) of ∼−65 mV was used. Thus, at V_h_ = −65mV, Cl^−^-mediated inhibitory postsynaptic currents (IPSCs) were nearly invisible, and cation-mediated excitatory postsynaptic currents (EPSCs) are inward. To isolate single vesicular events, 500 nM TTX was applied, and the frequency and amplitude of EPSCs in TTX were monitored. To assess whole-cell glutamate receptor–mediated currents, cells were whole-cell voltage-clamped at −40mV and recorded in the presence of the GABA_A_R antagonist Gabazine (10μM, Tocris 1262). N-methyl-D-aspartic acid (NMDA, 10 μM; Tocris 0114) or kainic acid (Tocris, 1 μM; Tocris 0222) were bath applied sequentially (ensuring that holding current returned to the original control level before subsequent drug application, 10 to 15 min), and the resulting change in current was measured. Peak shifts in holding current induced by each drug were reported as the appropriate glutamate receptor–mediated current.

To study inhibitory synapses, pipettes were filled with internal solutions containing the following (in millimolar): 140 K-gluconate, 1.4 NaCl, 1.5 MgCl_2,_ 0.5 CaCl_2_, 10 HEPES, 0.2 EGTA, 4 MgATP, and 0.5 Na_2_GTP (pH 7.2 to 7.3). E_Cl_ calculated for these solutions was ∼−85mV, and a holding potential (V_h_) of −50mV was used. Thus, at V_h_= −50mV, Cl^−^-mediated IPSCs are outward, and cation-mediated EPSCs are inward. All drugs were dissolved in aCSF and bath applied. To isolate single vesicular events, 500 nM TTX was applied, and the frequency and amplitude of IPSCs in the TTX were monitored.

### Electrophysiology Data Analysis.

The frequencies and amplitudes of spontaneous and miniature EPSCs and IPSCs (sEPSCs, sIPSCs mEPSCs, and mIPSCs, respectively) were measured using automatic detection of these events with Mini Analysis 6.0.7 (Synaptosoft) software followed by inspection of individual events for analysis. For assessment of synaptic current frequencies, events during the 180 s immediately before TTX application (defined as the baseline period) were compared to mean frequencies observed from 100 s after TTX application onset (over 180 s). The change of mean current induced by NMDA and kainic acid was calculated by defining the mean current in 20-s segments for control solution and at the peak of the NMDA and kainic acid applications by making histograms of all data points and then fitting a Gaussian distribution to each histogram to define the mean current (using Clampfit 10.4; Molecular Devices).

### Statistics.

**Fig. 1.**
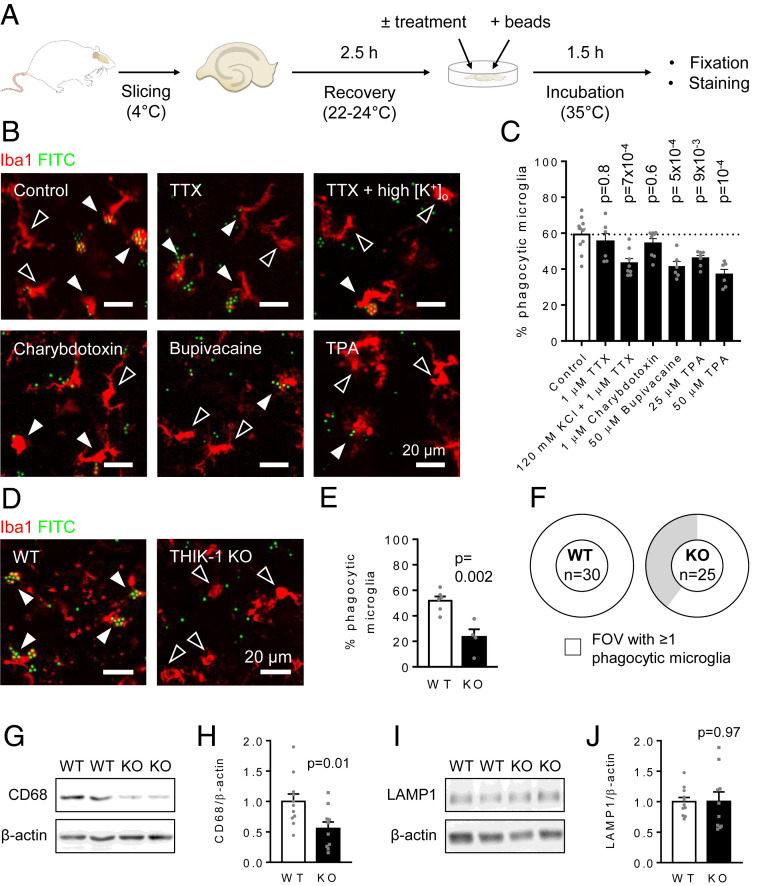
THIK-1 channels regulate microglial phagocytosis. (*A*) Diagram of the phagocytosis assay. (*B*) Representative single-plane images of microglia (Iba1, red) in acute hippocampal rat slices incubated with 3-µm microbeads (FITC, green), either untreated (control) or treated with 1 µM TTX, 1 µM TTX + 120 mM KCl (high [K^+^]_o_), 1 µM charybdotoxin, 50 µM bupivacaine, or 50 µM TPA. Black arrowheads indicate nonphagocytic cells, and white arrowheads indicate phagocytic cells. Some microbeads remain outside microglia. (*C*) Percentage of microglia that phagocytosed microbeads in each condition, showing a reduction by high [K^+^]_o_ and two-pore K^+^ channel block (bupivacaine, TPA) but not by calcium-activated K^+^ channel block (charybdotoxin) (control: *n* = 26 slices from 10 animals; TTX: nine slices from six animals; TTX + 120 mM KCl: 15 slices from eight animals; charybdotoxin: 14 slices from eight animals; bupivacaine: nine slices from six animals; 25 µM TPA: 19 slices from six animals; 50 µM TPA: 15 slices from five animals; and animals were used as the statistical unit). *P* values compare with control and are corrected for multiple comparisons. (*D*) Representative single-plane images of microglia in acute hippocampal slices from WT and THIK-1 KO mice incubated with 3-µm microbeads. (*E*) Percentage of microglia that phagocytosed microbeads in each genotype, showing a reduction in the KO. (*F*) Doughnut charts showing that only 60% of the analyzed fields of view (FOV) contained phagocytic microglia in the KO, while all did in the WT. The number of total microglia per FOV was not different (WT: 15.0 ± 1.8, KO: 12.3 ± 2.1; and *P* = 0.34) between genotypes. (*G*) Western blot for the microglial lysosomal marker CD68 in radioimmunoprecipitation assay–soluble homogenates from hippocampi of P17 WT or THIK-1 KO mice (β-actin was loading control). (*H*) Densitometric quantification showing THIK-1 KO reduces CD68 level (WT: *n* = 11 animals; KO: *n* = 10 animals). (*I* and* J*) As in *G* and *H* but for the broadly expressed lysosomal marker LAMP1.

**Fig. 2.**
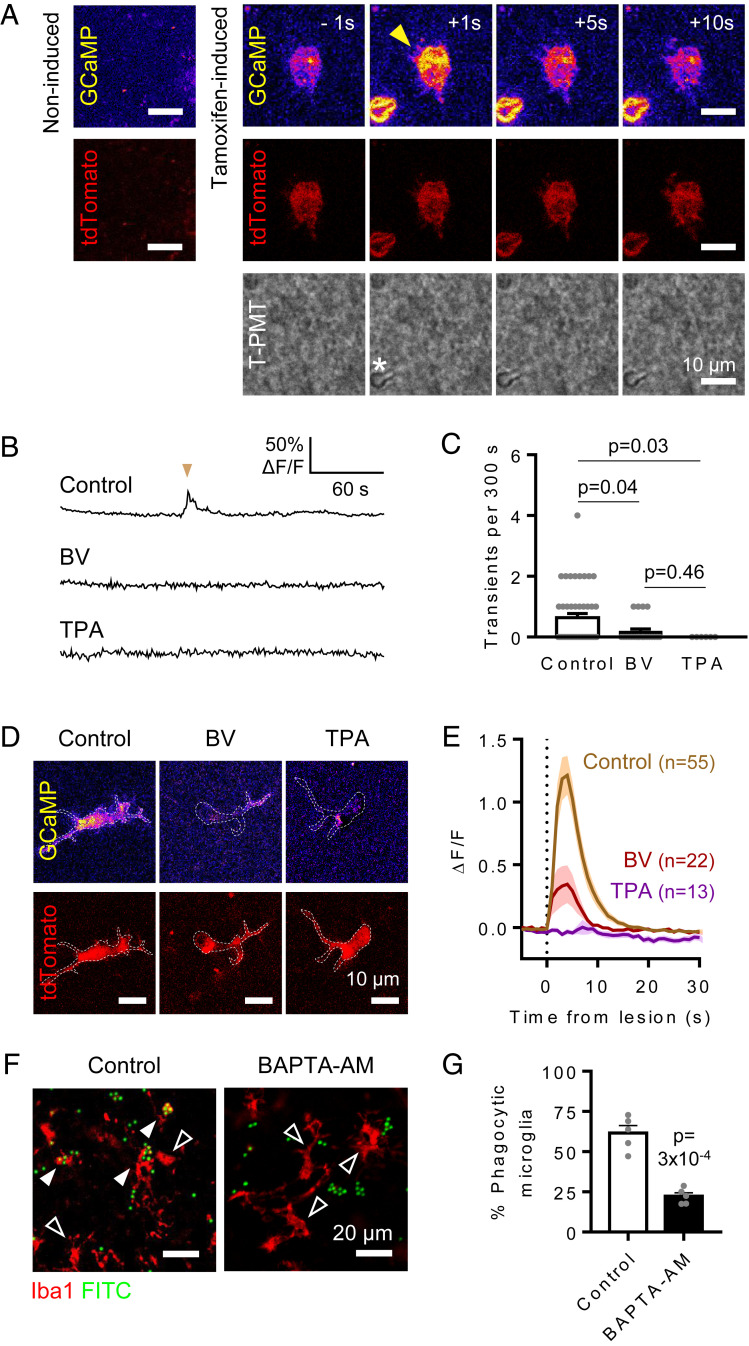
Two-pore domain K^+^ channels regulate Ca^2+^ activity in microglia. (*A*) Tamoxifen-induced microglia from *Cx3cr1*^CreER^ × GCaMP5g-IRES-tdTomato mice expressed the calcium reporter GCaMP5g (yellow) and tdTomato (red), which were not detected in brain slices from noninduced animals (*Left*). Acute laser lesion (star in the transmitted light channel, T-PMT) evokes a rapid [Ca^2+^]_I_ rise in tdTomato-labeled microglia (yellow arrowhead). Times indicate seconds from laser ablation. (*B* and* C*) Spontaneous Ca^2+^ transients in GCaMP5g-expressing microglia occur less often in cells treated with 50 µM bupivacaine (BV) or 50 µM TPA compared to control cells, as shown by the representative traces for GCaMP fluorescence changes (ΔF/F) (*B*) and transient rates over 5 min (*C*). (*D*) Representative microglial cells incubated in the absence (control) or presence of 50 µM BV or 50 µM TPA (ΔF/F for GCaMP5g shown at peak). (*E*) [Ca^2+^]_i_ levels over time showing increase upon laser lesion (vertical dashed line). TPA (*n* = 13 from 3 mice; *P* = 10^−4^) and BV (*n* = 22 cells from 4 mice; *P* = 3 × 10^−3^) significantly reduced the lesion-induced [Ca^2+^]_i_ rise compared to control (*n* = 56 from eight mice). No significant difference was found between BV and TPA (*P* = 0.2). (*F*) Representative single-plane images of microglia (Iba1, red) in acute hippocampal rat slices incubated with 3-µm microbeads (FITC, green) in the absence (control) or presence of 50 µM BAPTA-AM to chelate intracellular calcium. Black arrowheads indicate nonphagocytic cells, and white arrowheads indicate phagocytic cells. (*G*) Percentage of microglia that phagocytosed microbeads in each condition, showing a significant reduction by BAPTA-AM (eight slices from five animals per condition; animals were used as the statistical unit).

Data are presented as mean ± SEM. Data normality was assessed using the D'Agostino–Pearson test. Statistical significance (taken as *P* < 0.05) was assessed using unpaired two-tailed Student’s *t* tests (Figs. 1 *E*, *H*, and *J*, 2*G*, 3 *B*–*G* and *I*, and 4 *C*, *F*, and *G* and *SI Appendix*, Figs. S4*D* and S8 *B*, *C*, and *E*), Mann–Whitney *U* tests (Figs. 4 *B*, *D*, and *E* and *SI Appendix*, Figs. S3, S4*B*, S5–S7, and S8*D*), or one-way ANOVA followed by Dunnett’s post hoc tests for individual comparisons (Figs. 1*C* and 2 *C* and *E* and *SI Appendix*, Fig. S2). All statistical analysis was performed in Microsoft Excel 2016, GraphPad Prism 7, and Sigmaplot 11.

**Fig. 3.**
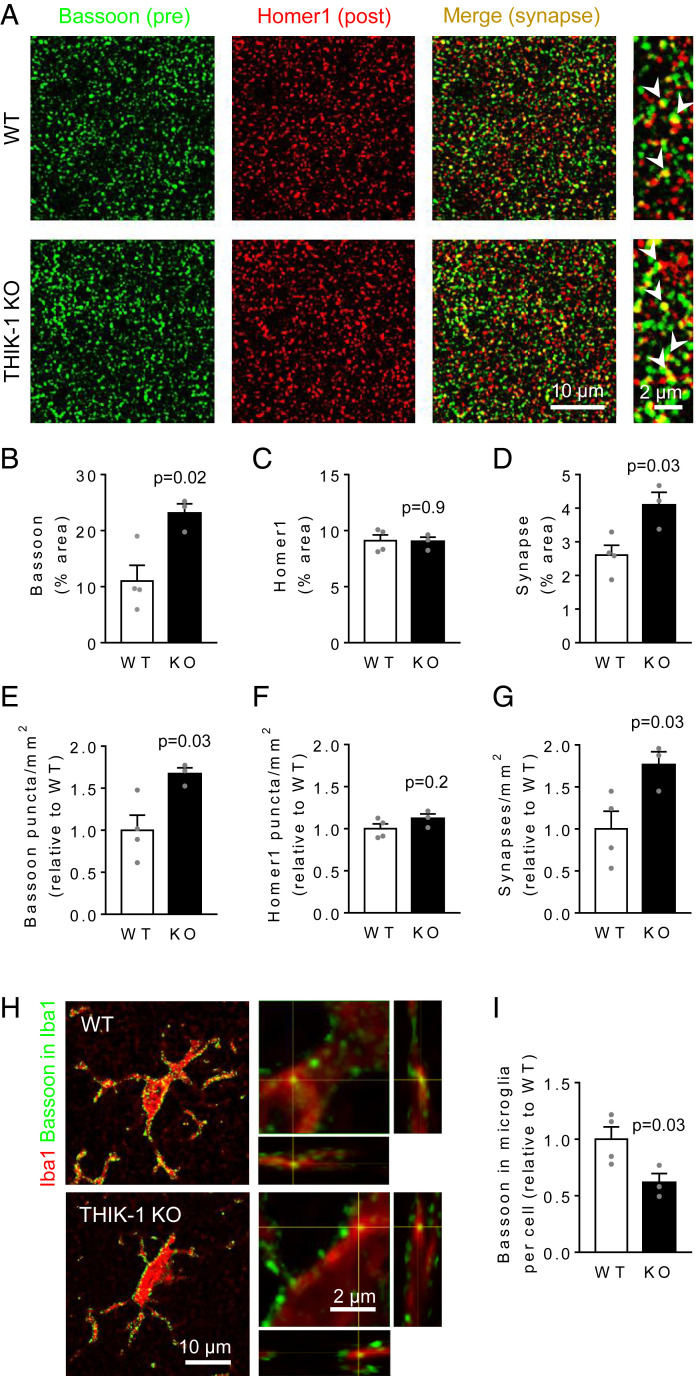
Microglial phagocytosis of presynaptic material is reduced by THIK-1 KO. (*A*) Representative confocal images from the CA1 stratum radiatum of WT or THIK-1 KO mice at P17 to P19, showing the presynaptic marker Bassoon (green) and the excitatory postsynaptic marker Homer1 (red). The merged image and expanded views on the right show colocalized puncta (yellow). (*B*–*D*) Quantification of the area covered by (*B*) Bassoon, (*C*) Homer1, and (*D*) colocalized puncta, showing an increased fraction of the imaged area labeled for synapses in KO mice. (*E*–*G*) Quantification of the numbers of (*E*) Bassoon, (*F*) Homer, and (*G*) synaptic puncta per square millimeter, showing increased numbers of presynaptic puncta and synapses in KO mice. Average numbers in WT mice were 53 puncta/100 µm^2^ for Bassoon, 42 puncta/100 µm^2^ for Homer1, and 26 synapses/100 µm^2^. (For *B*–*G*, WT: *n* = 4 animals; KO: *n*=3 animals; and three confocal stacks from five brain slices averaged per animal.) (*H*) Representative confocal images showing Bassoon puncta (green) located within microglia (Iba1, red). On the right, close-up of microglial processes with orthogonal projections at the level of the crosshairs showing Bassoon puncta within microglia. (*I*) Quantification of the area of Bassoon puncta colocalizing with each microglial cell, showing a decrease in KO microglia (WT: 20 cells from four animals; KO: 15 cells from three animals; and animals were used as the statistical unit).

**Fig. 4.**
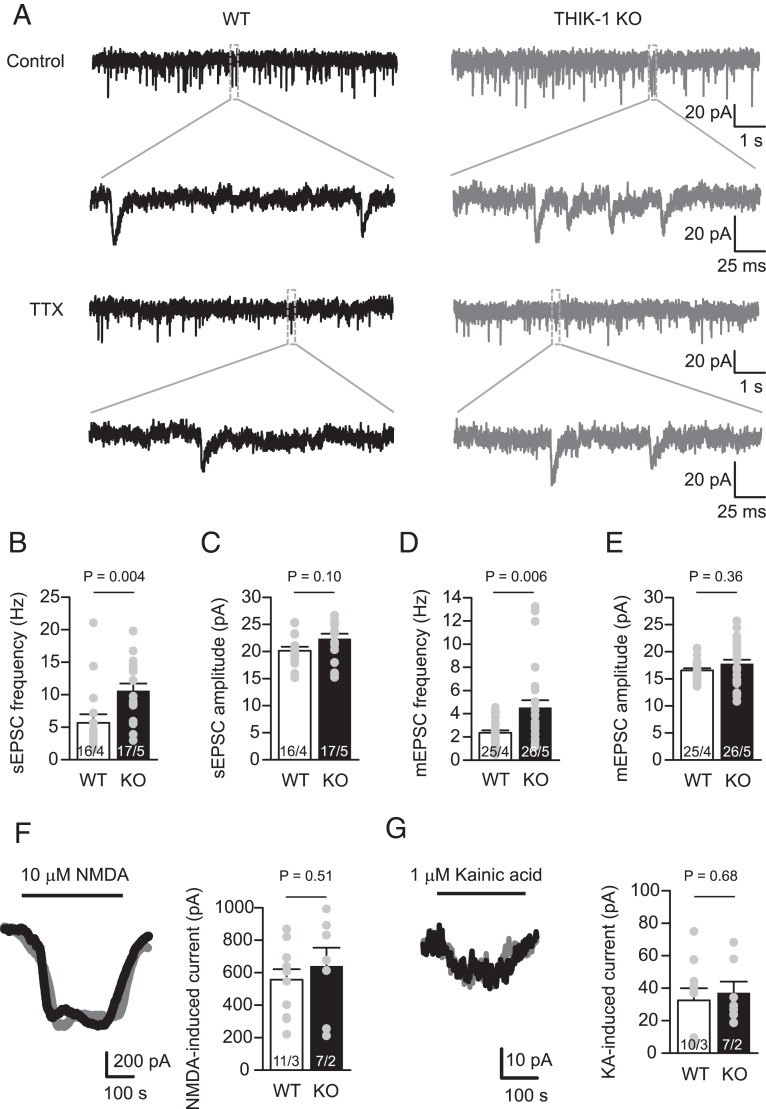
THIK-1 KO increases excitatory synaptic transmission. (*A*) Representative current traces from whole-cell voltage-clamped CA1 pyramidal neurons (V_h_ = −65 mV, E_Cl_ = −62mV) in WT (black, left) and THIK-1 KO (gray, right) hippocampal slices. The top two rows show spontaneous EPSCs (sEPSCs) (i.e., including both miniature EPSCs [mEPSCs] and those evoked by spontaneous action potentials). The bottom two rows show mEPSCs in 500 nM TTX. The second and fourth row traces expand the indicated areas in the first and third rows. (*B* and* C*) Bar graphs showing sEPSC (*B*) frequency and (*C*) amplitude, comparing WT and THIK-1 KO. Overlaid gray circles show raw values in individual cells. Numbers on bars are of cells/animals, and SEM values use cells as the statistical unit. (*D* and* E*) As for *C* and *D* but showing mEPSC (*D*) frequency and (*E*) amplitude, comparing WT and THIK-1 KO in 500 nM TTX. (*F*) Representative traces of whole-cell currents (V_h_ = −40 mV, E_Cl_ = −62 mV) from WT (black) and THIK-1 KO (red) hippocampal CA1 pyramidal cells and bar graph of mean current induced by 10 µM NMDA bath application to WT and THIK-1 KO cells. (*G*) As for *F* but applying 1 µM kainic acid.

## Results

### Microglial Phagocytosis Is Regulated by K^+^ Channels.

Ion channels and receptors controlling microglial motility might be involved in phagocytosis. These include P2Y_12_ receptors that regulate ATP-evoked microglial chemotaxis to an injury site (35) and THIK-1 K^+^ channels that regulate microglial morphology and surveillance (3). It was previously reported that P2Y_12_ receptors regulate phagocytosis (36, 37), but the role of microglial K^+^ channels is unknown. Therefore, we first tested whether blocking THIK-1 (the dominant K^+^ channel expressed in “resting” microglia) affects microglial phagocytosis. We studied phagocytosis in situ in brain slices, because microglia in slices retain their normal morphology and interactions with surrounding cells, which they lose in vitro, and critically, cultured microglia rapidly change their protein expression profiles (38) and do not express THIK-1 (*Kcnk13*) K^+^ channels (39), which are the main channels setting the resting potential in microglia in vivo (3). Following a recovery period of a few hours after brain slicing, which allows some microglial activation to occur (29), fluorophore-labeled resin microbeads were applied (30, 31) onto rat hippocampal slices in the presence or absence of pharmacological blockers (Fig. 1*A*).

Microglia engulf and phagocytose their substrates thanks to membrane protrusions and phagocytic cups, the formation of which relies upon Ca^2+^-dependent cytoskeletal rearrangements (40–42). Indeed, microglia were seen to form phagocytic cups and engulf microbeads in situ (*SI Appendix*, Fig. S1). As negative control experiments, the brain slices were incubated at 4 °C or in the presence of the actin inhibitor cytochalasin D, both ofwhich disrupt cytoskeletal dynamics (43). We found that both conditions potently blocked phagocytosis (*SI Appendix*, Fig. S2). MRS2578, a blocker of the P2Y_6_ receptor known to regulate microglial phagocytosis (44, 45), also reduced the fraction of microglia that was phagocytic (*SI Appendix*, Fig. S2).

Potassium efflux across the microglial membrane via THIK-1 was previously found to control NLRP3 activation (3, 46). We found that elevating the extracellular K^+^ concentration ([K^+^]_o_) to prevent such efflux and depolarize the cells reduced the fraction of microglia that were phagocytic by a third (Fig. 1 *B* and*C*). This was done in the presence of TTX to block voltage-gated Na^+^ channels and thus action potential–evoked synaptic transmitter release from neurons (TTX had no effect by itself: Fig. 1*C*). To determine how the raised [K^+^]_o_ altered phagocytosis, we applied blockers of different K^+^ channel types. Blocking Ca^2+^-activated K^+^ channels with charybdotoxin had no significant effect, but both the K^+^ channel blockers bupivacaine and tetrapentylammonium (TPA) inhibited phagocytosis (Fig. 1 *B* and *C*). Since bupivacaine blocks both two-pore domain channels and voltage-gated Na^+^ channels while TPA blocks both two-pore domain channels and voltage-gated K^+^ channels (47, 48), these data are consistent with K^+^ efflux via THIK-1 (or downstream changes in microglial membrane voltage, V_m_) regulating phagocytosis. In addition to pharmacology, we also useda mouse in which THIK-1 was knocked out (KO) and found that deletion of THIK-1 had a similar inhibitory effect on microglial phagocytosis (Fig. 1 *D*–*F*). Differences may arise between chemically blocking a channel and deleting it genetically (which may have a stronger effect when the chemical block is not complete but may also suffer from compensatory changes). In addition, species differences (i.e., between rat and mouse) may play a role. However, no significant difference was found between the decrease in phagocytic ability induced by chemical and genetic removal of THIK-1 currents (one-way ANOVA, *P* = 0.2).

Lastly, we assessed whether protein levels of lysosome markers were affected in the THIK-1 KO mice (since phagocytosed particles are ultimately passed to lysosomes for digestion). Levels of the microglial lysosome marker CD68 were approximately halved in hippocampal homogenates from KO mice (Fig. 1 *G* and *H*), while the homologous lysosome marker LAMP1, which is also expressed by other brain cells including astrocytes and oligodendrocytes (49), remained unchanged (Fig. 1 *I* and *J*), indicating a specific effect of THIK-1 KO on the lysosomes that degrade material phagocytosed by microglia.

### THIK-1 Controls Ca^2+^ Activity in Microglia.

Intracellular calcium concentration ([Ca^2+^]_i_) has been suggested to regulate phagocytosis (41, 42, 44, 50). To further understand how THIK-1 controls microglial phagocytic ability, we imaged Ca^2+^ in microglia from *Cx3cr1*^CreER^ × GCaMP5g-IRES-tdTomato mice, which express the genetically encoded Ca^2+^ indicator GCaMP5g in a tamoxifen-inducible manner (Fig. 2*A*). Phagocytic cells exhibit periodic spontaneous Ca^2+^ transients as well as stimulus-evoked [Ca^2+^]_i_ rises (51). Consistently with previous work (52), microglia showed a low frequency of spontaneous Ca^2+^ activity in brain slices (∼0.13 transients/min; Fig. 2 *B* and *C*). Blocking THIK-1 channels with 50 µM bupivacaine or 50 µM TPA significantly decreased the frequency of these events (Fig. 2 *B* and *C*). In addition, focal laser lesions were used as a well-established proxy to evoke Ca^2+^ responses in microglia. Lesions triggered a rapid Ca^2+^ rise (Fig. 2*A*), in agreement with previous reports (53). While untreated microglia exhibited consistent evoked Ca^2+^ responses, block of THIK-1 currents reduced this response (Fig. 2 *D* and *E*).

Buffering of intracellular calcium concentration with 50 µM BAPTA-AM (a membrane-permeable ester form of BAPTA) reduced microglial phagocytic rate by 75% (Fig. 2 *F* and *G*), supporting the idea that Ca^2+^ is required for microglial phagocytosis. Thus, reduction of Ca^2+^ transient activity by THIK-1 may provide a mechanism to explain its role in microglial phagocytosis.

### THIK-1 Regulates Microglial Phagocytosis of Synapses.

Deficits in microglial phagocytosis could result in impaired pruning of synapses during development (54), so we tested the effects of THIK-1 KO on hippocampal synapse numbers. Using P17 to P19 mice, when synapse pruning in the hippocampus is near its peak (12, 13), we assessed the labeling of presynaptic (Bassoon) and postsynaptic glutamatergic (Homer1) markers in the stratum radiatum of the CA1 hippocampal region by immunohistochemistry (33). A colocalization of both markers was taken to indicate an excitatory synapse (Fig. 3*A*). In the THIK-1 KO, while no postsynaptic change was detected, the fraction of the imaged area labeled by the presynaptic marker approximately doubled compared to that in wild-type littermates (WT). As a result, the derived total synaptic area was higher in KO mice (Fig. 3 *B*–*D*). The increase in colocalization was produced by a 67% increase in the number (Fig. 3 *E*–*G*) but not the size (*SI Appendix*, Fig. S3) of presynaptic terminals, with no change in the number of postsynaptic terminals (Fig. 3*F*).

To demonstrate that the increase in presynaptic terminal number was caused by a reduction of phagocytosis, we next assessed the presence of Bassoon puncta inside Iba1-labeled microglia (Fig. 3*H*). In THIK-1 KO mice, Bassoon colocalization with microglia was significantly reduced (Fig. 3*I*). By contrast, Homer1 colocalization with microglia was not altered in the KO (*SI Appendix*, Fig. S4 *A* and *B*) and the density of dendritic spines was not affected either (*SI Appendix*, Fig. S4 *C* and *D*). Taken together, these data suggest that THIK-1 regulates the number of glutamatergic synapses by promoting microglial uptake specifically of presynaptic material.

### THIK-1 Regulates Excitatory Synaptic Transmission.

To further confirm that THIK-1 regulates removal of functional excitatory synapses, we performed whole-cell voltage-clamp recording of CA1 pyramidal neurons from P17 to P19 mice (Fig. 4). We found that the spontaneous EPSC frequency was enhanced in pyramidal neurons from THIK-1 KO compared to WT mice, with no amplitude alteration (Fig. 4 *A*–*C*). Since this increase in the KO could be due either to a higher number of excitatory synapses or to higher activity (or an increased vesicle release probability) of presynaptic neurons, we bath-applied TTX to block action potential–mediated neurotransmitter release. Consistent with our immunohistochemical studies showing more synapses in the KO (Fig. 3), mEPSC frequency approximately doubled in THIK-1 KO pyramidal neurons compared to in WT cells, without an amplitude change (Fig. 4 *A*,* D*, and* E*).

This effect of THIK-1 deletion on synapse levels did not result from an altered number of microglia, as overall microglial density and distribution in CA1 were similar between THIK-1 WT and KO mice (*SI Appendix*, Fig. S5). Furthermore, we found that THIK-1 only regulates excitatory synapses, with no detectable effect on inhibitory synapses. Neither the frequencies nor amplitudes of sIPSCs or mIPSCs were affected by THIK-1 KO (*SI Appendix*, Fig. S6). Altogether, our data suggest that THIK-1 deficiency leads to an increase in the number of functional excitatory synapses, which is due to THIK-1 regulating microglial internalization of excitatory presynaptic terminals, presumably via its effects on the microglial membrane potential and/or [Ca^2+^]_i_ transients.

We tested postsynaptic effects of THIK-1 deficiency electrophysiologically (Fig. 4 *F* and *G*) by bath-applying the glutamate receptor agonists NMDA (to activate NMDA receptors, 10 µM; Fig. 4*F*) and kainic acid (to activate AMPA/KA receptors, 1 µM; Fig. 4*G*). Consistent with our staining showing no effect on postsynaptic terminals (Fig. 3*C* and *SI Appendix*, Fig. S4), we found no significant differences between the THIK-1 KO and WT in their NMDA- and kainate-induced macroscopic currents (Fig. 4 *F* and *G*). Thus, THIK-1 deficiency selectively enhances the number of excitatory presynaptic release sites without affecting the postsynaptic glutamate receptor density (assessed from the spontaneous and miniature EPSC amplitudes) or the total (synaptic plus extrasynaptic) glutamate receptor density (assessed from the response to NMDA and kainate). Interestingly, the effect of THIK-1 deletion on excitatory synapse number was transient during development, as no changes in synapse number (*SI Appendix*, Fig. S7) or synaptic transmission (*SI Appendix*, Fig. S8) were detected between adult WT and KO mice. This supports the notion of a key developmental role for THIK-1-mediated regulation of synapse number, which may also become reactivated in disease.

## Discussion

Microglia are not merely passive support cells. Instead, they continuously survey the brain parenchyma and control neuronal function. THIK-1 channels, the main K^+^ channels in “resting” microglia, regulate their ramification, surveillance, and cytokine release (3). Here, using pharmacology and THIK-1 KO mice, we demonstrated that microglial phagocytosis is also controlled by THIK-1 (Fig. 1).

The requirement of THIK-1 for phagocytosis may in part be due to its role in enhancing microglial ramification and surveillance, which will increase the probability of a microglial cell encountering a target to phagocytose. However, the tonically active THIK-1 may also promote phagocytosis by keeping microglia hyperpolarized (3), and this hyperpolarization will increase the driving force for Ca^2+^ entry. Indeed, a hyperpolarized membrane voltage is associated with phagocytosis in macrophages (20), while depolarization is seen in TREM2 KOmicroglia in which phagocytosis is reduced (21). Furthermore, we found that THIK-1 block reduced spontaneous and damage-evoked Ca^2+^ transient activity in microglia (Fig. 2 *A*–*E*), which may be required for phagocytosis (41, 42, 44, 50), consistent with our demonstration that buffering [Ca^2+^]_i_ reduced microglial phagocytosis (Fig. 2 *F* and* G*). A need for calcium concentration changes in phagocytosis and the fact that THIK-1 KO reduces such changes provide a possible mechanism for how THIK-1 KO inhibits phagocytosis. Protein levels of the microglial lysosome marker CD68 were also reduced in the THIK-1 KO (Fig. 1 *G* and* H*). These data suggest that there is a specific effect of THIK-1 KO on phagocytosis and downstream lysosomal degradation, although we cannot rule out a contribution by the decreased surveillance in microglia lacking THIK-1.

THIK-1 is expressed both in microglia and oligodendrocytes (49). In the absence of a microglial-selective THIK-1 KO mouse being available, four independent results indicate that the effects of global THIK-1 KO on synapse number are mediated by microglial changes. First, THIK-1 KO roughly halved the number of microglia which phagocytose fluorescent microbeads (Fig. 1*E*). Second, we show that THIK-1 KO reduced by 40% the amount of the presynaptic protein Bassoon that was internalized into microglia (Fig. 3 *H* and *I*). Third, microglia are the only phagocytic cell type expressing THIK-1 channels in the brain (49) as oligodendrocytes reportedly lack phagocytic machinery (55). Finally, the protein level of a microglial lysosomal marker (CD68) was approximately halved in theTHIK-1 KO hippocampus, while the level of a lysosomal marker (LAMP1) expressed by other brain cells (including astrocytes and oligodendrocytes) was unchanged (Fig. 1 *G*–*J*). These data are consistent with a direct role for THIK-1 in controlling phagocytosis by microglia specifically, although indirect signaling to microglia via oligodendrocytes (where this channel is also expressed) cannot be excluded.

As a result of impaired phagocytic ability in THIK-1 KO microglia, the number of hippocampal excitatory synapses was increased in these mice during development, as shown both by immunolabeling and electrophysiology (Figs. 3 and 4). We found that THIK-1-mediated promotion of synapse loss was mainly an effect on the number of presynaptic terminals (Fig. 3 *E*–*G*). While others have reported internalization of postsynaptic material as well (13, 56), there is now evidence that microglial phagocytosis preferentially targets presynaptic compartments both in health (12, 14) and disease (25, 26, 57). This might be partly due to “eat-me” tags (such as C1q) (58) being preferentially located on presynaptic sites. Here, reported changes in excitatory synapse number (Fig. 3) and synaptic transmission (Fig. 4) reflect the net effect of THIK-1 on synapse formation and removal. While we directly assess engulfment of synaptic material by microglia (Fig. 3 *H* and *I* and *SI Appendix*, Fig. S4), an additional contribution of THIK-1 to synapse formation—which may be induced by microglial contact (11, 12)—cannot be excluded.

On the other hand, we found that deleting THIK-1 had no effect on inhibitory synapses (*SI Appendix*, Fig. S6), suggesting that phagocytosis by microglia mainly targets excitatory synapses, and thus that there is a difference in the recognition molecules expressed on excitatory versus inhibitory synapses. Indeed, microglial depletion increases mEPSC frequency in brain slices (59) and in vivo (60), while mIPSCs remain unaltered (60).

In line with reports that microglial phagocytosis may affect synapse numbers transiently during development (7, 13), we found that THIK-1 deletion did not affect synapse numbers in healthy adult mice (*SI Appendix*, Fig. S7). This suggests that another mechanism operating in parallel can, on a long time scale, correct the number of synapses present. Nevertheless, developmental pruning mechanisms are activated again in disease scenarios after the normal developmental period is over, which can be detrimental if synapse removal is excessive (61).

In fact, synapse loss is a strong indicator of cognitive decline (62–64). Synaptic deficits precede amyloid deposition in animal models of dementia (65), and microglial phagocytosis of synaptic material is increased in Alzheimer’s patients (66). Ablation of microglia rescues synaptic loss and reduces memory impairment in mouse models of dementia (67). Thus, manipulating microglia–synapse interactions may provide clinical benefit for conditions causing cognitive impairment. Specifically, being able to block microglial phagocytosis in a time-controlled manner could help protect synapses from removal. As human microglia express *Kcnk13*, the gene encoding THIK-1 (68, 69), a role of microglial THIK-1 in regulating human synapse turnover is conceivable. It would be interesting to examine whether short-term block of THIK-1 in live human brain slices (e.g., using THIK-1-blocking anesthetics such as isoflurane or sevoflurane) increases synapse number, and the magnitude and duration of any such effect. Microglial responses (and especially phagocytosis) are crucial for the development and progression of dementia (70). Since amyloid-targeting therapies for Alzheimer’s disease have largely failed, possibly because therapeutic interventions are given too late (62), it would be advantageous to devise therapeutic agents that control phagocytosis to prevent synapse loss early on.

## Data Availability

Code for analyzing microglial properties and calcium concentration changes data have been deposited in GitHub (https://github.com/AttwellLab). All other study data are included in the article and/or *SI Appendix*.
